# Supplementary figures and images for: Friend leukemia integration 1 overexpression decreases endometrial receptivity and induces embryo implantation failure by promoting PART1 transcription in the endometrial epithelial cells
Source: PeerJ. 2023 Sep 26;11:e16105. doi: 10.7717/peerj.16105 (PMC10540769; doi:10.7717/peerj.16105)

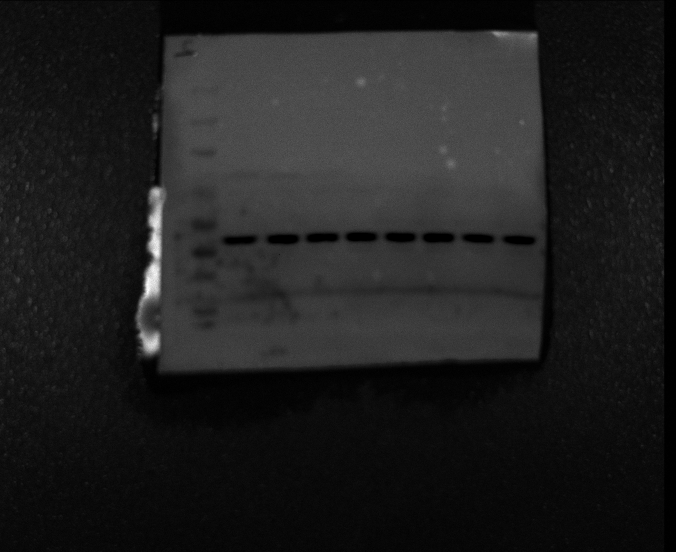

Supplement: Supplemental Information 2 [file peerj-11-16105-s002.zip › uncropped wb blots/Figure 2/b-actin.tif]

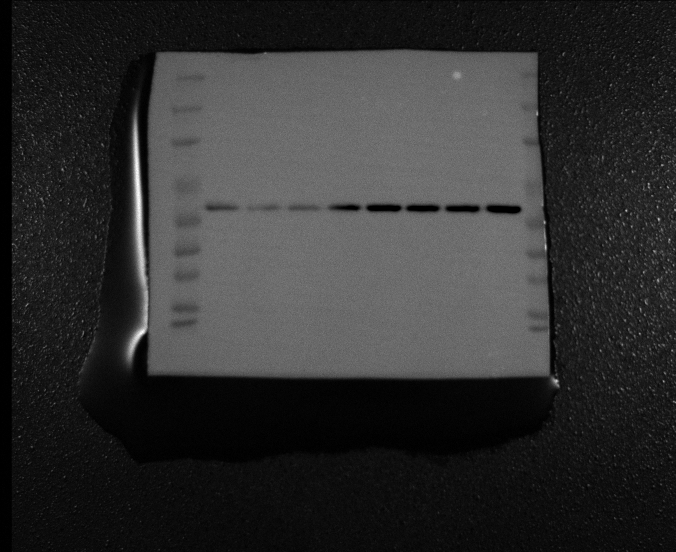

Supplement: Supplemental Information 2 [file peerj-11-16105-s002.zip › uncropped wb blots/Figure 2/FLI1.tif]

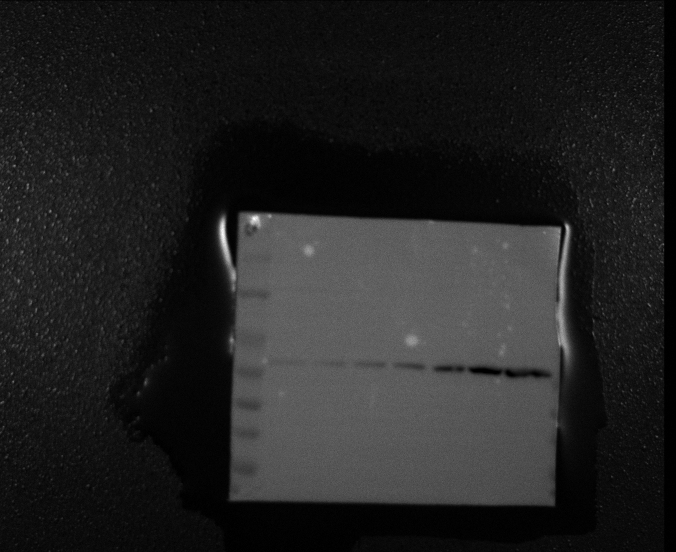

Supplement: Supplemental Information 2 [file peerj-11-16105-s002.zip › uncropped wb blots/Figure 4/FIL1.tif]

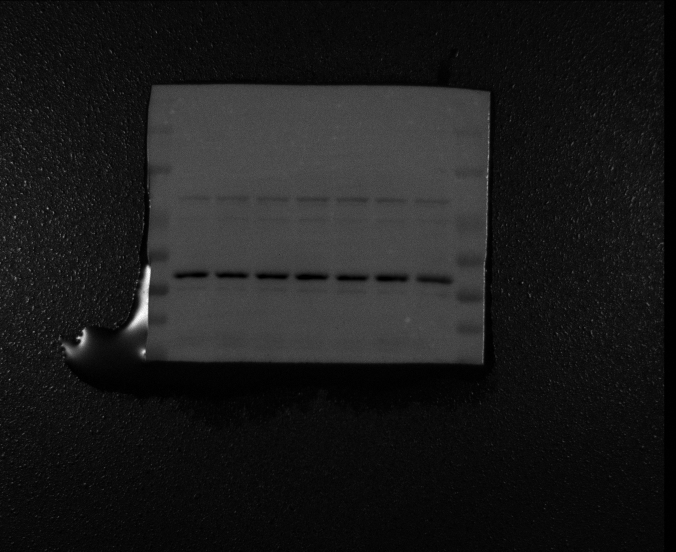

Supplement: Supplemental Information 2 [file peerj-11-16105-s002.zip › uncropped wb blots/Figure 4/b-actin.tif]

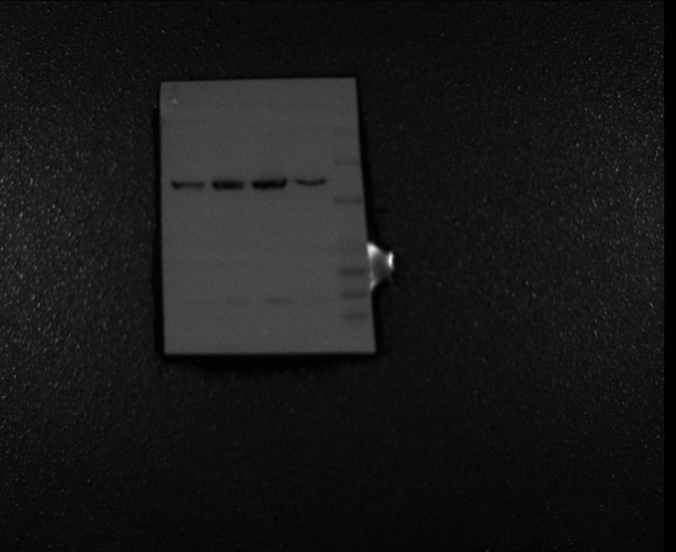

Supplement: Supplemental Information 2 [file peerj-11-16105-s002.zip › uncropped wb blots/Figure 7/E-cadherin.tif]

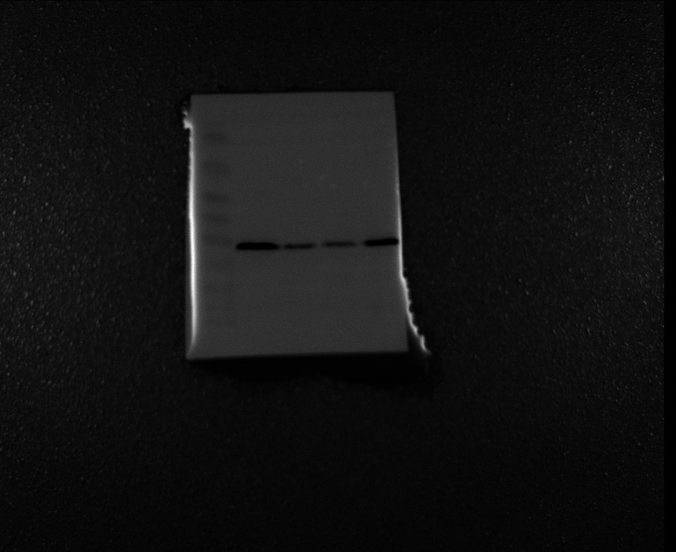

Supplement: Supplemental Information 2 [file peerj-11-16105-s002.zip › uncropped wb blots/Figure 7/vimentin.tif]

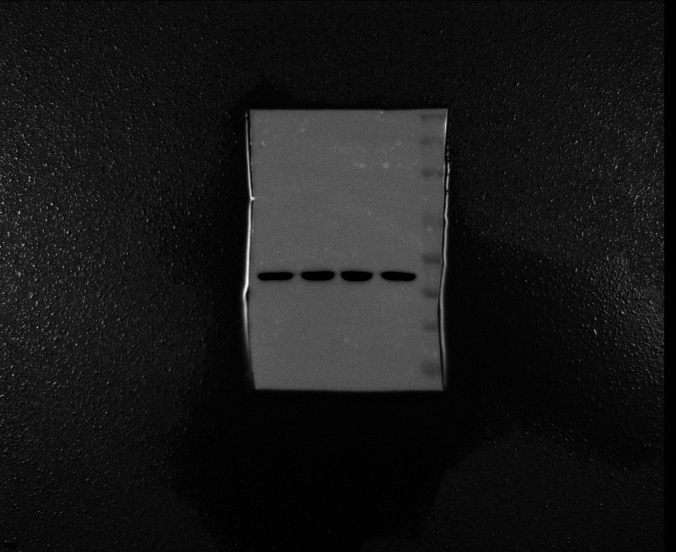

Supplement: Supplemental Information 2 [file peerj-11-16105-s002.zip › uncropped wb blots/Figure 7/b-actin.tif]

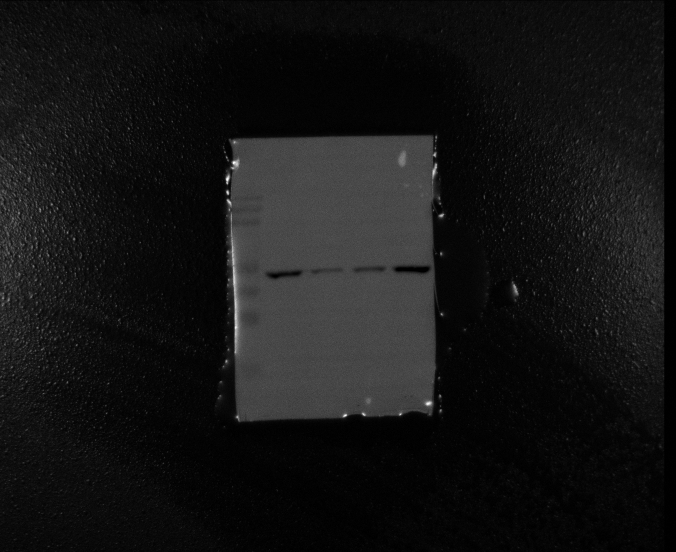

Supplement: Supplemental Information 2 [file peerj-11-16105-s002.zip › uncropped wb blots/Figure 3/Figure 3C/FLI 1.tif]

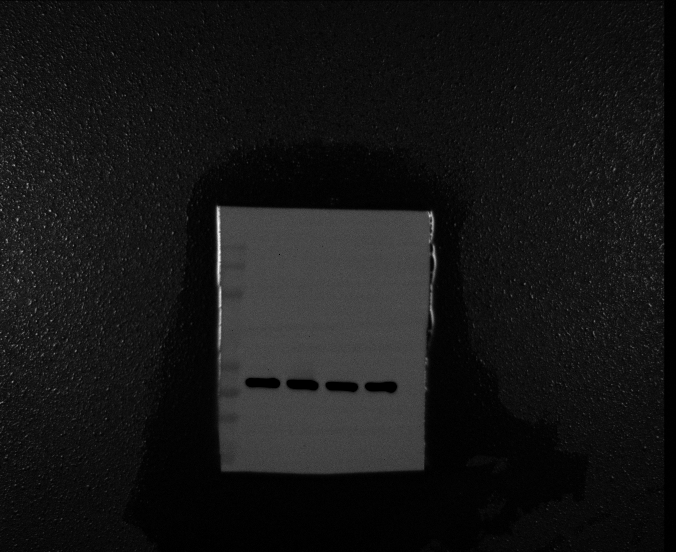

Supplement: Supplemental Information 2 [file peerj-11-16105-s002.zip › uncropped wb blots/Figure 3/Figure 3C/b-actin.tif]

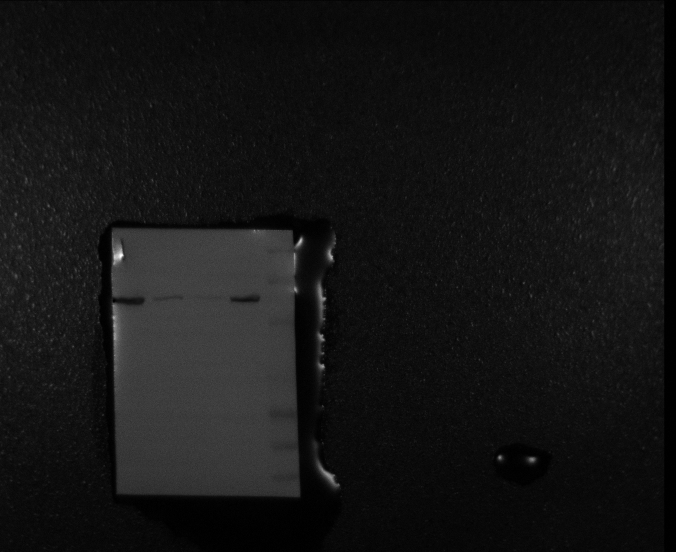

Supplement: Supplemental Information 2 [file peerj-11-16105-s002.zip › uncropped wb blots/Figure 3/Figure 3G/E-cadherin.tif]

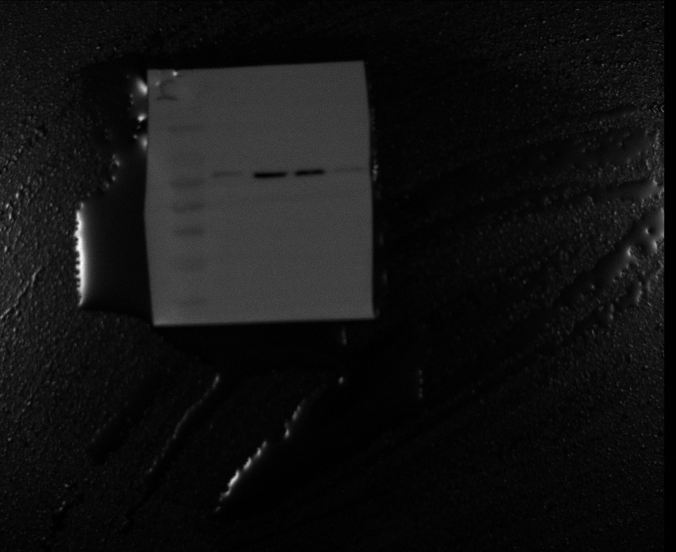

Supplement: Supplemental Information 2 [file peerj-11-16105-s002.zip › uncropped wb blots/Figure 3/Figure 3G/Vimentin.tif]

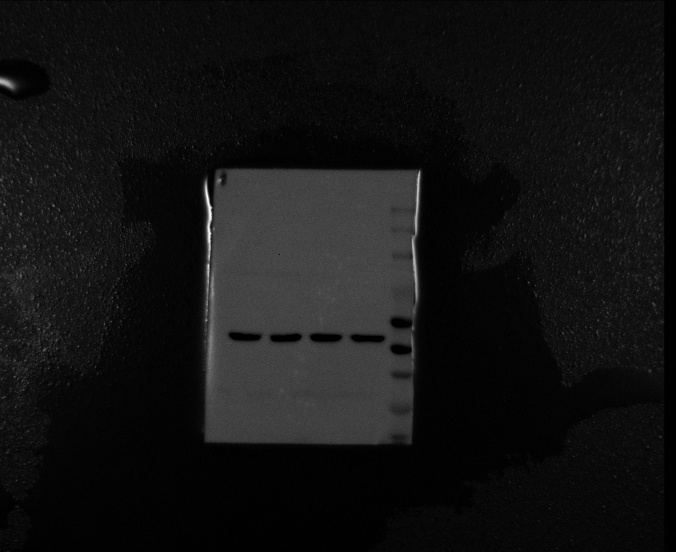

Supplement: Supplemental Information 2 [file peerj-11-16105-s002.zip › uncropped wb blots/Figure 3/Figure 3G/b-actin.tif]

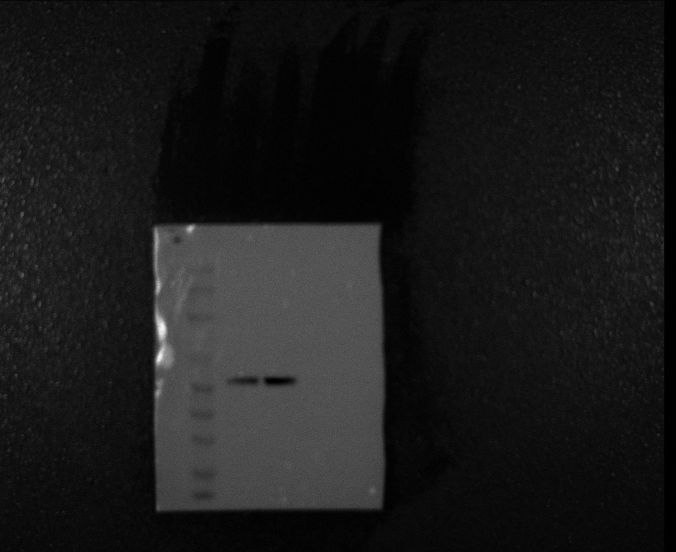

Supplement: Supplemental Information 2 [file peerj-11-16105-s002.zip › uncropped wb blots/Figure 6/FLI1.tif]

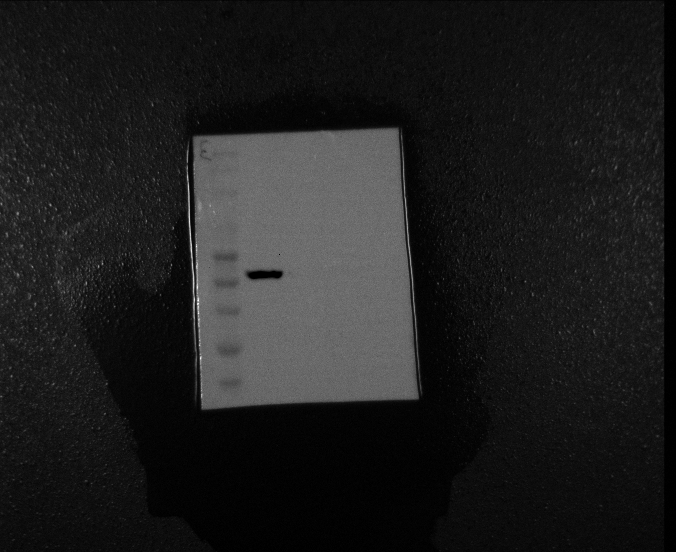

Supplement: Supplemental Information 2 [file peerj-11-16105-s002.zip › uncropped wb blots/Figure 6/β-actin.tif]
