# Supplementary material for: Friend leukemia integration 1 overexpression decreases endometrial receptivity and induces embryo implantation failure by promoting PART1 transcription in the endometrial epithelial cells
Source: PeerJ. 2023 Sep 26;11:e16105. doi: 10.7717/peerj.16105 (PMC10540769; doi:10.7717/peerj.16105)
Supplement: Supplemental Information 3 [file peerj-11-16105-s003.pdf]

Figure 2D

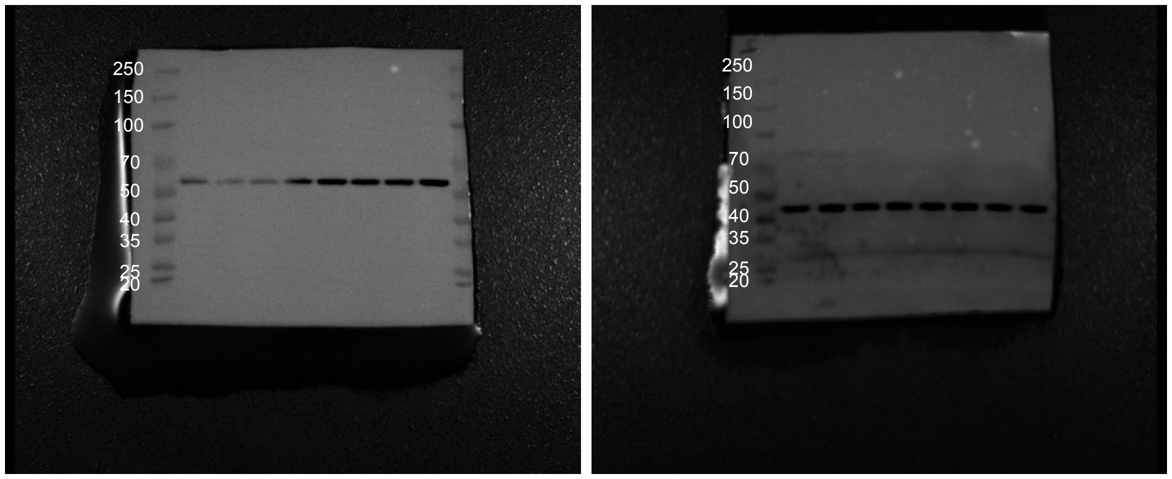

FLI1 β-actin

Figure 3C

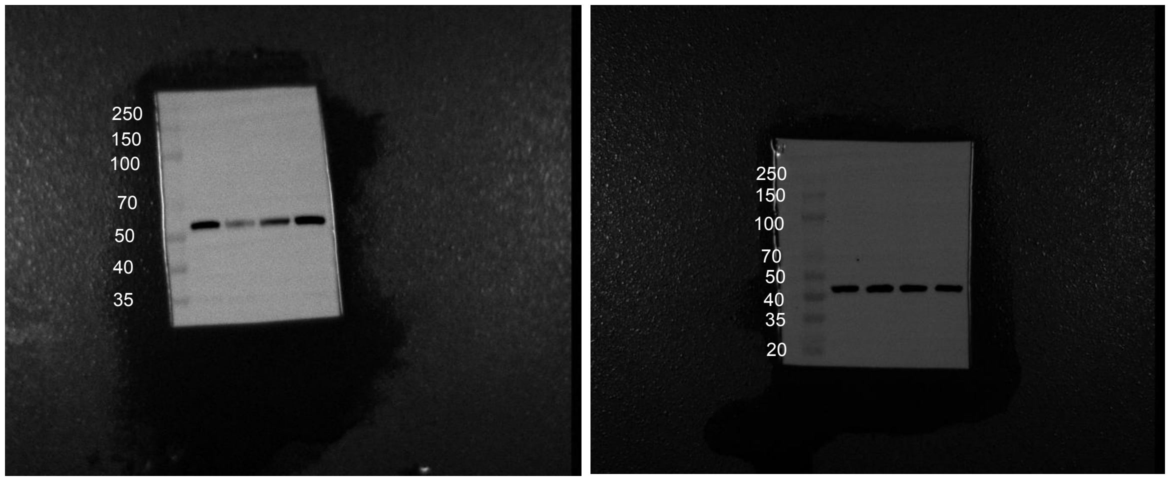

FLI1 β-actin

Figure 3G

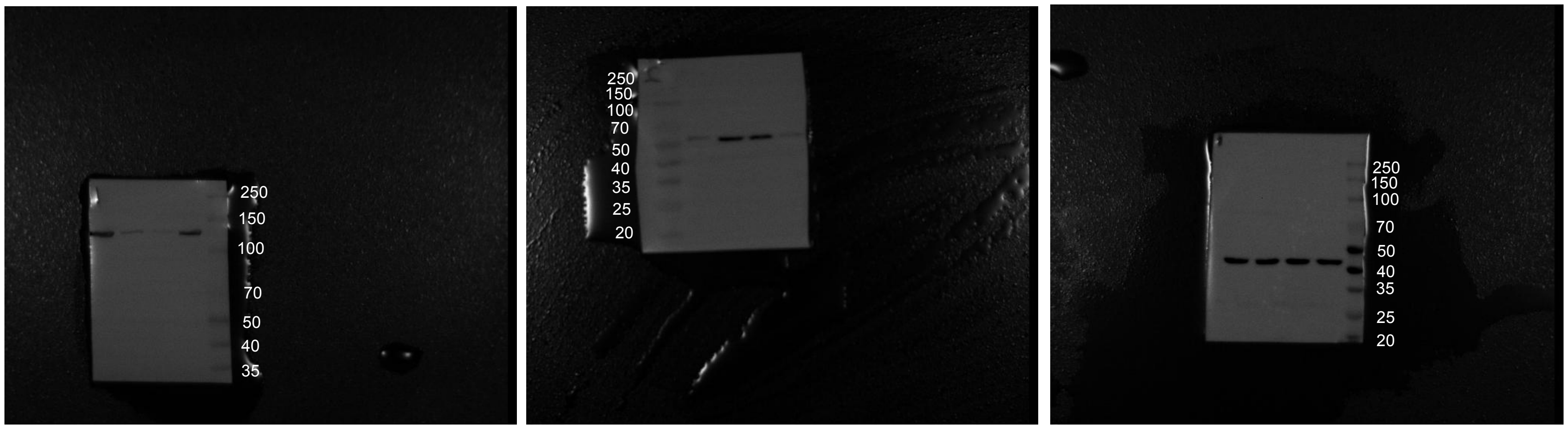

E-Cadherin Vimentin β-actin

Figure 4B

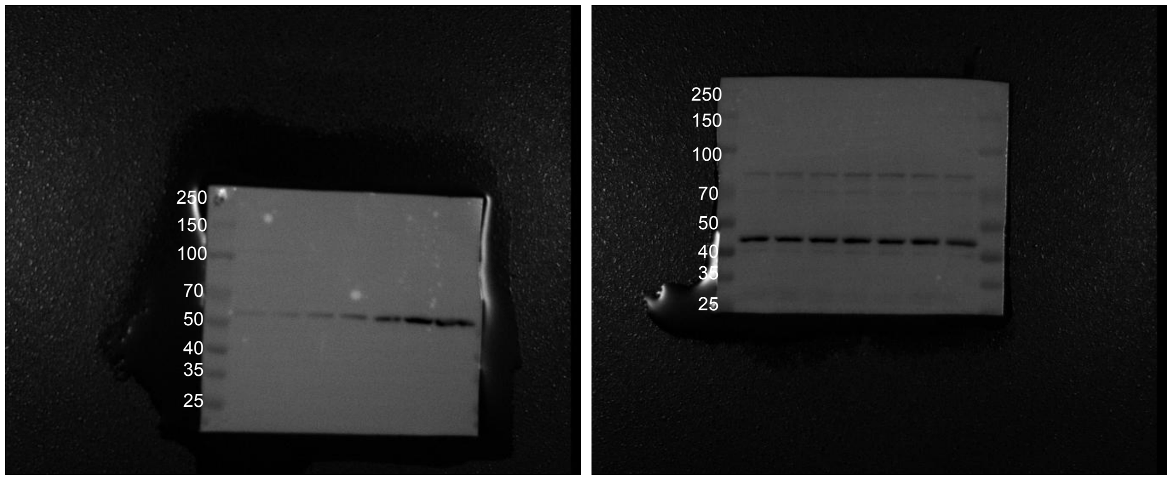

FLI1 β-actin

Figure 6D

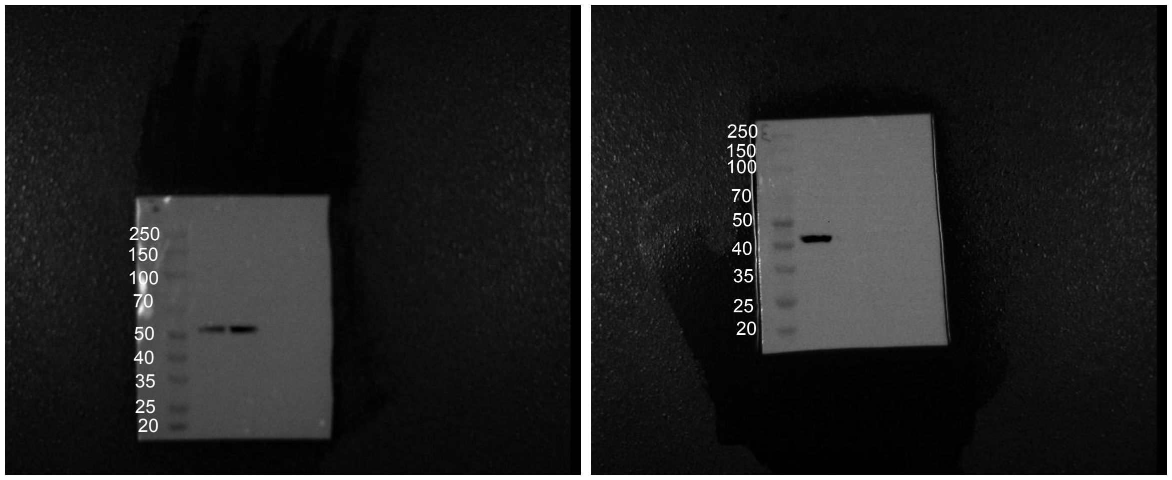

FLI1 β-actin

Figure 7D

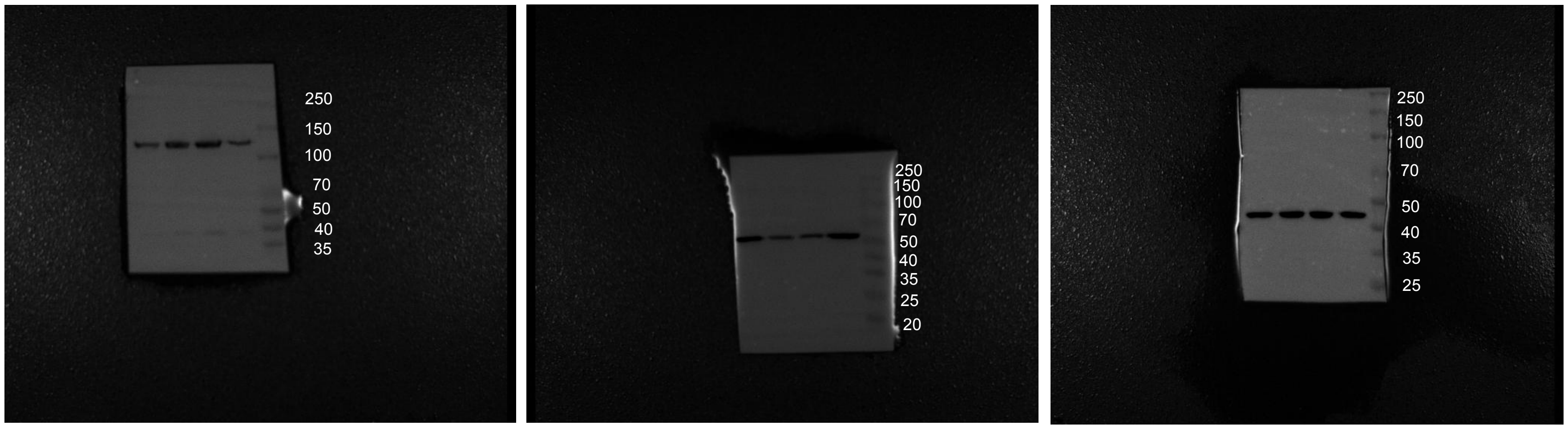

E-Cadherin Vimentin β-actin
